# 3-[(*E*)-(Benzyl­iminiumyl)meth­yl]-6,8-di­chloro-2*H*-chromen-4-olate

**DOI:** 10.1107/S1600536813018072

**Published:** 2013-07-10

**Authors:** Yoshinobu Ishikawa, Yuya Motohashi

**Affiliations:** a School of Pharmaceutical Sciences, University of Shizuoka, 52-1 Yada, Suruga-ku, Shizuoka 422-8526, Japan

## Abstract

In the title compound, C_17_H_13_Cl_2_NO_2_, the H atom of the –OH group is transferred to the N atom of the imine, forming a zwitterion. Thus, there is a intra­molecular O⋯H—N, rather than O—H⋯N, hydrogen bond, which forms a six-membered ring.

## Related literature

For the biological propertries of similar structures, see: Khan *et al.* (2009 ▶); Tu *et al.* (2013 ▶). For related structures, see: Benaouida *et al.* (2013 ▶); Małecka & Budzisz (2006 ▶).
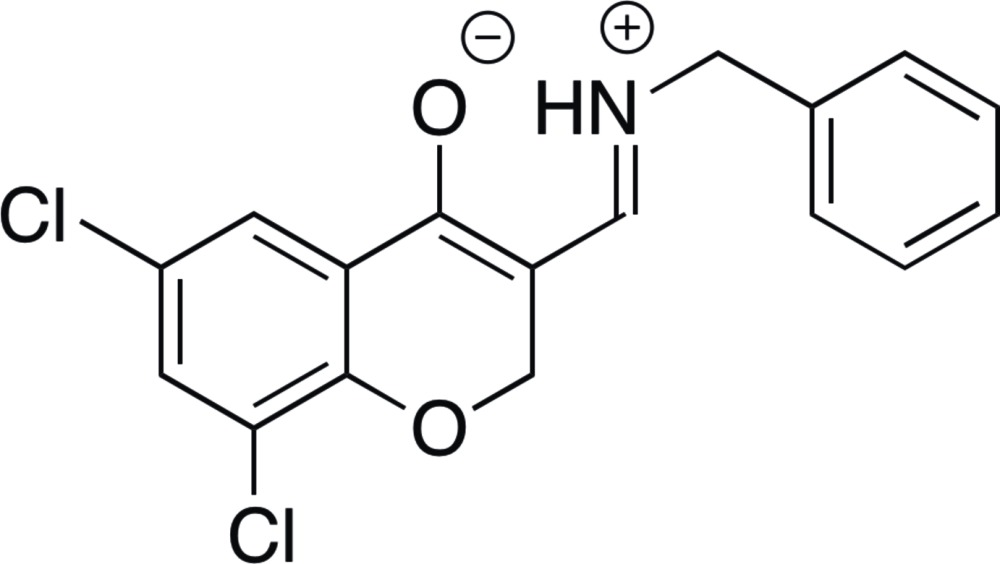


## Experimental

### 

#### Crystal data

C_17_H_13_Cl_2_NO_2_
*M* *_r_* = 334.20
Orthorhombic, 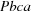

*a* = 11.312 (5) Å
*b* = 28.284 (8) Å
*c* = 9.294 (4) Å
*V* = 2973.7 (19) Å^3^
*Z* = 8
Mo *K*α radiation
μ = 0.44 mm^−1^
*T* = 100 K
0.40 × 0.40 × 0.30 mm

#### Data collection

Rigaku AFC7R diffractometer
Absorption correction: ψ scan (North *et al.*, 1968 ▶) *T*_min_ = 0.734, *T*_max_ = 0.876
4921 measured reflections
3411 independent reflections
2842 reflections with *F* ^2^ > 2.0σ(*F* ^2^)
*R*_int_ = 0.027
3 standard reflections every 150 reflections intensity decay: −0.7%

#### Refinement

*R*[*F* ^2^ > 2σ(*F* ^2^)] = 0.034
*wR*(*F* ^2^) = 0.095
*S* = 1.01
3411 reflections
199 parameters
H-atom parameters constrained
Δρ_max_ = 0.37 e Å^−3^
Δρ_min_ = −0.30 e Å^−3^

### 

Data collection: *WinAFC* (Rigaku, 1999 ▶); cell refinement: *WinAFC*; data reduction: *WinAFC*; program(s) used to solve structure: *SHELXS97* (Sheldrick, 2008 ▶); program(s) used to refine structure: *SHELXL97* (Sheldrick, 2008 ▶); molecular graphics: *CrystalStructure* (Rigaku, 2010 ▶); software used to prepare material for publication: *CrystalStructure*.

## Supplementary Material

Crystal structure: contains datablock(s) global, I. DOI: 10.1107/S1600536813018072/rn2117sup1.cif

Structure factors: contains datablock(s) I. DOI: 10.1107/S1600536813018072/rn2117Isup2.hkl

Additional supplementary materials:  crystallographic information; 3D view; checkCIF report

## Figures and Tables

**Table 1:** Hydrogen-bond geometry (Å, °)

*D*—H⋯*A*	*D*—H	H⋯*A*	*D*⋯*A*	*D*—H⋯*A*
N5—H5⋯O4	0.88	2.15	2.7737 (19)	127

**Fig. 1.**
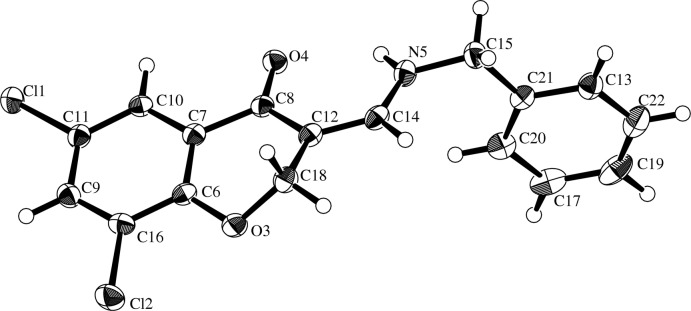
The molecular structure of the title compound, with displacement ellipsoids drawn at the 50% probability level. Hydrogen atoms are shown as small spheres of arbitrary radius.

## supplementary crystallographic information

### Comment

Schiff bases of 3-formyl chromones have attracted much attention due to their biological functions such as enzyme inhibition (Khan *et al.* 2009; Tu *et al.* 2013). Here we report the crystal structure of the title compound, which was obtained from the condensation reaction of 6,8-dichloro-3-formylchromone with benzylamine and successive reduction by use of 2-picoline borane. The structure shows that the H atom of the –OH group is transferred to the N5 atom of the imine, thus forming a zwitterion. As a result, an intramolecular O···H–N [O···N = 2.7737 (19) Å], rather than O–H···N, hydrogen bond is formed. The bond distances O4–C8 [1.251 (2) Å], C8–C12 [1.432 (3) Å], C12–C14 [1.376 (3) Å] and C14–N5 [1.328 (3) Å] and torsion angles O4–C8–C12–C14 [–2.0 (3)°] and C8–C12–C14–N5 [0.6 (3)°] in the six-membered ring indicate charge delocalization among the atoms. This effect might be responsible for the preferential reduction of the α,β-unsaturated carbonyl of the synthetic intermediate, rather than reduction of the imine.

### Experimental

Benzylamine (5 mmol), 6,8-dichloro-3-formylchromone (5 mmol) and 2-picoline borane (5 mmol) were dissolved in a mixture of MeOH-AcOH (10:1, 60 ml) and 2-propanol (20 ml), and stirred overnight at room temperature. Hydrochloric acid (1 M, 20 ml) was added to the reaction mixture, which was then stirred for 30 min. After neutralization with saturated NaHCO_3_, the mixture was extracted with methylene chloride. The extract was washed with brine, dried over anhydrous Na_2_SO_4_ and purified by column chromatography on silica gel (*n*-hexane: ethyl acetate = 3: 1). The eluted fractions were concentrated and filtered off. Layering *n*-hexane on the filtrate gave single crystals suitable for X-ray diffraction (yield 6%).

### Refinement

The carbon-bound hydrogen atoms were placed in geometrical positions [C–H 0.95 to 0.99 Å, *U*_iso_(H) = 1.2*U*_eq_(C)], and refined using a riding model. The hydrogen atom of the OH group was found to be located near N5 of the imine in a difference Fourier map, and refined with distance restraint [N–H 0.88 Å, *U*_iso_(H) = 1.2*U*_eq_(N)].

### Crystal data

**Table:**

C_17_H_13_Cl_2_NO_2_	*F*(000) = 1376.00
*M**_r_* = 334.20	*D*_x_ = 1.493 Mg m^−^^3^
Orthorhombic, *P**b**c**a*	Mo *K*α radiation, λ = 0.71069 Å
Hall symbol: -P 2ac 2ab	Cell parameters from 25 reflections
*a* = 11.312 (5) Å	θ = 15.3–17.4°
*b* = 28.284 (8) Å	µ = 0.44 mm^−^^1^
*c* = 9.294 (4) Å	*T* = 100 K
*V* = 2973.7 (19) Å^3^	Prismatic, yellow
*Z* = 8	0.40 × 0.40 × 0.30 mm

### Data collection

**Table:**

Rigaku AFC7R diffractometer	*R*_int_ = 0.027
ω scans	θ_max_ = 27.5°
Absorption correction: ψ scan (North *et al.*, 1968)	*h* = −8→14
*T*_min_ = 0.734, *T*_max_ = 0.876	*k* = 0→36
4921 measured reflections	*l* = −12→6
3411 independent reflections	3 standard reflections every 150 reflections
2842 reflections with *F*^2^ > 2.0σ(*F*^2^)	intensity decay: −0.7%

### Refinement

**Table:**

Refinement on *F*^2^	Secondary atom site location: difference Fourier map
*R*[*F*^2^ > 2σ(*F*^2^)] = 0.034	Hydrogen site location: inferred from neighbouring sites
*wR*(*F*^2^) = 0.095	H-atom parameters constrained
*S* = 1.01	*w* = 1/[σ^2^(*F*_o_^2^) + (0.0484*P*)^2^ + 1.5727*P*] where *P* = (*F*_o_^2^ + 2*F*_c_^2^)/3
3411 reflections	(Δ/σ)_max_ = 0.001
199 parameters	Δρ_max_ = 0.37 e Å^−^^3^
0 restraints	Δρ_min_ = −0.30 e Å^−^^3^
Primary atom site location: structure-invariant direct methods	

### Special details

**Table:**

Refinement. Refinement was performed using all reflections. The weighted *R*-factor (*wR*) and goodness of fit (*S*) are based on *F*^2^. *R*-factor (gt) are based on *F*. The threshold expression of *F*^2^ > 2.0 σ(*F*^2^) is used only for calculating *R*-factor (gt).

### Fractional atomic coordinates and isotropic or equivalent isotropic displacement parameters (Å^2^)

**Table:**

	*x*	*y*	*z*	*U*_iso_*/*U*_eq_	
Cl1	0.35615 (4)	0.728872 (14)	0.62619 (5)	0.03107 (12)	
Cl2	0.08088 (4)	0.696080 (16)	0.16439 (5)	0.03462 (13)	
O3	0.15191 (10)	0.59834 (4)	0.20309 (12)	0.0270 (3)	
O4	0.39867 (10)	0.54598 (4)	0.48567 (12)	0.0250 (3)	
N5	0.35872 (12)	0.45877 (5)	0.35813 (15)	0.0254 (3)	
C6	0.19963 (14)	0.62709 (5)	0.30470 (16)	0.0225 (3)	
C7	0.27643 (13)	0.61039 (5)	0.41191 (16)	0.0207 (3)	
C8	0.31307 (14)	0.55972 (5)	0.41132 (16)	0.0214 (3)	
C9	0.22286 (14)	0.70675 (6)	0.39517 (17)	0.0250 (4)	
C10	0.32453 (14)	0.64196 (5)	0.51118 (16)	0.0214 (3)	
C11	0.29724 (14)	0.68935 (5)	0.50180 (17)	0.0230 (4)	
C12	0.24579 (14)	0.53004 (6)	0.31714 (17)	0.0232 (4)	
C13	0.45884 (15)	0.34680 (6)	0.17648 (18)	0.0281 (4)	
C14	0.27199 (14)	0.48304 (6)	0.29639 (17)	0.0245 (4)	
C15	0.37317 (15)	0.40812 (6)	0.33640 (18)	0.0266 (4)	
C16	0.17522 (14)	0.67549 (6)	0.29714 (17)	0.0245 (4)	
C17	0.58227 (15)	0.41186 (8)	0.01394 (19)	0.0354 (5)	
C18	0.13558 (14)	0.55016 (6)	0.25306 (19)	0.0262 (4)	
C19	0.59128 (16)	0.36417 (8)	−0.0169 (2)	0.0383 (5)	
C20	0.51113 (15)	0.42726 (6)	0.12718 (18)	0.0286 (4)	
C21	0.44961 (13)	0.39468 (6)	0.20942 (17)	0.0226 (4)	
C22	0.52919 (17)	0.33175 (7)	0.0642 (2)	0.0360 (5)	
H5	0.4091	0.4738	0.4140	0.0305*	
H9	0.2052	0.7395	0.3899	0.0300*	
H10	0.3758	0.6309	0.5848	0.0257*	
H13	0.4163	0.3243	0.2319	0.0337*	
H14	0.2230	0.4662	0.2311	0.0294*	
H15A	0.2940	0.3938	0.3233	0.0319*	
H15B	0.4081	0.3943	0.4245	0.0319*	
H17	0.6247	0.4342	−0.0422	0.0425*	
H18A	0.1104	0.5301	0.1713	0.0315*	
H18B	0.0718	0.5495	0.3260	0.0315*	
H19	0.6402	0.3538	−0.0937	0.0459*	
H20	0.5049	0.4601	0.1479	0.0344*	
H22	0.5349	0.2990	0.0426	0.0433*	

### Atomic displacement parameters (Å^2^)

**Table:**

	*U*^11^	*U*^22^	*U*^33^	*U*^12^	*U*^13^	*U*^23^
Cl1	0.0410 (3)	0.0228 (2)	0.0294 (3)	−0.00185 (17)	−0.00490 (18)	−0.00298 (15)
Cl2	0.0342 (3)	0.0345 (3)	0.0352 (3)	0.00614 (17)	−0.01121 (18)	0.00487 (18)
O3	0.0296 (6)	0.0275 (6)	0.0239 (6)	−0.0012 (5)	−0.0059 (5)	−0.0005 (5)
O4	0.0252 (6)	0.0244 (6)	0.0255 (6)	0.0005 (5)	−0.0039 (5)	0.0005 (5)
N5	0.0264 (7)	0.0227 (7)	0.0272 (7)	−0.0027 (6)	0.0003 (6)	−0.0041 (6)
C6	0.0212 (8)	0.0271 (8)	0.0192 (7)	−0.0011 (6)	0.0023 (6)	−0.0004 (6)
C7	0.0193 (7)	0.0227 (8)	0.0202 (7)	−0.0019 (6)	0.0033 (6)	0.0004 (6)
C8	0.0222 (8)	0.0230 (7)	0.0191 (7)	−0.0011 (6)	0.0036 (6)	0.0008 (6)
C9	0.0257 (8)	0.0228 (8)	0.0265 (8)	0.0020 (7)	0.0049 (7)	0.0025 (7)
C10	0.0210 (8)	0.0239 (8)	0.0193 (7)	−0.0013 (6)	0.0015 (6)	0.0013 (6)
C11	0.0238 (8)	0.0230 (8)	0.0222 (8)	−0.0023 (6)	0.0033 (7)	−0.0013 (6)
C12	0.0225 (8)	0.0260 (8)	0.0210 (8)	−0.0019 (7)	0.0015 (7)	−0.0016 (6)
C13	0.0261 (8)	0.0300 (9)	0.0281 (9)	0.0022 (7)	−0.0025 (7)	−0.0038 (7)
C14	0.0235 (8)	0.0285 (8)	0.0215 (8)	−0.0035 (7)	0.0034 (7)	−0.0031 (6)
C15	0.0284 (9)	0.0219 (8)	0.0295 (9)	−0.0015 (7)	0.0053 (7)	−0.0014 (7)
C16	0.0221 (8)	0.0287 (8)	0.0226 (8)	0.0027 (7)	0.0002 (7)	0.0052 (6)
C17	0.0203 (9)	0.0581 (12)	0.0278 (9)	−0.0083 (8)	0.0005 (7)	0.0030 (9)
C18	0.0248 (8)	0.0273 (8)	0.0267 (8)	−0.0026 (7)	−0.0024 (7)	−0.0032 (7)
C19	0.0223 (9)	0.0652 (14)	0.0272 (9)	0.0032 (9)	0.0012 (7)	−0.0125 (9)
C20	0.0221 (8)	0.0345 (9)	0.0294 (9)	−0.0055 (7)	−0.0018 (7)	−0.0007 (7)
C21	0.0168 (7)	0.0286 (8)	0.0225 (8)	−0.0007 (6)	−0.0017 (6)	−0.0027 (6)
C22	0.0321 (10)	0.0424 (10)	0.0337 (10)	0.0062 (8)	−0.0031 (8)	−0.0126 (8)

### Geometric parameters (Å, º)

**Table:**

Cl1—C11	1.7408 (17)	C15—C21	1.512 (3)
Cl2—C16	1.7322 (18)	C17—C19	1.383 (4)
O3—C6	1.3581 (19)	C17—C20	1.395 (3)
O3—C18	1.451 (2)	C19—C22	1.379 (3)
O4—C8	1.251 (2)	C20—C21	1.385 (3)
N5—C14	1.328 (3)	N5—H5	0.880
N5—C15	1.456 (3)	C9—H9	0.950
C6—C7	1.404 (3)	C10—H10	0.950
C6—C16	1.398 (3)	C13—H13	0.950
C7—C8	1.492 (2)	C14—H14	0.950
C7—C10	1.395 (2)	C15—H15A	0.990
C8—C12	1.432 (3)	C15—H15B	0.990
C9—C11	1.390 (3)	C17—H17	0.950
C9—C16	1.379 (3)	C18—H18A	0.990
C10—C11	1.378 (2)	C18—H18B	0.990
C12—C14	1.376 (3)	C19—H19	0.950
C12—C18	1.494 (3)	C20—H20	0.950
C13—C21	1.392 (3)	C22—H22	0.950
C13—C22	1.380 (3)		
			
Cl2···O3	2.9012 (14)	C6···H19^v^	2.7245
O3···C8	2.874 (2)	C7···H19^v^	3.2656
O4···N5	2.7737 (19)	C8···H14^vi^	3.0884
O4···C10	2.851 (2)	C8···H17^v^	3.5062
O4···C14	2.884 (2)	C8···H18A^vii^	3.5501
N5···C8	2.943 (3)	C9···H22^vi^	3.2255
N5···C20	2.894 (3)	C10···H13^vi^	3.5411
C6···C11	2.771 (3)	C10···H15A^vi^	3.3524
C6···C12	2.797 (3)	C10···H15B^ii^	3.2487
C7···C9	2.796 (3)	C11···H13^vi^	3.2488
C7···C18	2.761 (3)	C12···H17^v^	3.1140
C10···C16	2.777 (3)	C12···H20^iii^	3.3837
C13···C17	2.760 (3)	C13···H10^ii^	2.9701
C14···C20	3.504 (3)	C14···H17^v^	3.5246
C14···C21	3.307 (3)	C14···H17^iii^	3.1469
C16···C18	3.596 (3)	C14···H20^iii^	3.1337
C19···C21	2.781 (3)	C15···H10^ii^	3.1335
C20···C22	2.772 (3)	C15···H17^iii^	3.4792
Cl1···C9^i^	3.4408 (19)	C15···H18A^vi^	3.5736
Cl1···C13^ii^	3.5107 (19)	C16···H19^v^	2.9361
Cl1···C22^ii^	3.592 (3)	C16···H22^vi^	3.3726
Cl2···C11^iii^	3.566 (2)	C17···H14^vii^	3.2418
O3···N5^iv^	3.592 (3)	C17···H15A^vii^	2.8789
O3···C15^iv^	3.425 (3)	C17···H18B^iv^	2.6983
O3···C19^v^	3.544 (3)	C18···H5^iv^	3.2627
O4···O4^ii^	3.4773 (18)	C18···H15B^iv^	3.4691
O4···N5^ii^	3.108 (2)	C18···H17^vi^	3.5334
O4···C14^vi^	3.569 (3)	C18···H17^v^	3.3747
O4···C15^ii^	3.329 (3)	C18···H20^iii^	3.0867
O4···C18^vii^	3.481 (3)	C19···H15A^vii^	3.0330
N5···O3^vi^	3.592 (3)	C19···H18B^iv^	3.3900
N5···O4^ii^	3.108 (2)	C20···H10^ii^	3.3922
N5···C17^iii^	3.599 (3)	C20···H14^vii^	2.9477
C6···C19^v^	3.579 (3)	C20···H15A^vii^	3.3691
C9···Cl1^viii^	3.4408 (19)	C20···H18B^iv^	3.0242
C9···C22^vi^	3.433 (3)	C20···H18B^vii^	3.5530
C10···C13^vi^	3.569 (3)	C21···H10^ii^	2.8430
C11···Cl2^vii^	3.566 (2)	C22···H10^ii^	3.5942
C11···C13^vi^	3.475 (3)	H5···O3^vi^	3.4434
C13···Cl1^ii^	3.5107 (19)	H5···O4^ii^	2.4314
C13···C10^iv^	3.569 (3)	H5···C18^vi^	3.2627
C13···C11^iv^	3.475 (3)	H5···H5^ii^	2.9979
C14···O4^iv^	3.569 (3)	H5···H18A^vi^	2.4038
C14···C17^iii^	3.430 (3)	H5···H18A^vii^	2.8905
C14···C20^iii^	3.421 (3)	H5···H18B^vii^	3.5998
C15···O3^vi^	3.425 (3)	H9···Cl1^viii^	3.1181
C15···O4^ii^	3.329 (3)	H9···Cl2^i^	3.4352
C15···C17^iii^	3.574 (3)	H9···H13^x^	3.1291
C16···C22^vi^	3.398 (3)	H9···H22^vi^	3.2522
C17···N5^vii^	3.599 (3)	H9···H22^ix^	3.4450
C17···C14^vii^	3.430 (3)	H10···C13^ii^	2.9701
C17···C15^vii^	3.574 (3)	H10···C15^ii^	3.1335
C18···O4^iii^	3.481 (3)	H10···C20^ii^	3.3922
C19···O3^v^	3.544 (3)	H10···C21^ii^	2.8430
C19···C6^v^	3.579 (3)	H10···C22^ii^	3.5942
C20···C14^vii^	3.421 (3)	H10···H13^ii^	3.1695
C22···Cl1^ii^	3.592 (3)	H10···H14^vi^	3.2613
C22···C9^iv^	3.433 (3)	H10···H15A^vi^	3.0153
C22···C16^iv^	3.398 (3)	H10···H15B^ii^	2.5476
Cl1···H9	2.7981	H13···Cl1^iv^	3.5668
Cl1···H10	2.8069	H13···Cl1^ii^	3.2600
Cl2···H9	2.8075	H13···C10^iv^	3.5411
O4···H5	2.1512	H13···C11^iv^	3.2488
O4···H10	2.5850	H13···H9^xi^	3.1291
N5···H20	2.5602	H13···H10^ii^	3.1695
C6···H9	3.2783	H14···O4^iv^	2.6862
C6···H10	3.2801	H14···C8^iv^	3.0884
C6···H18A	3.1745	H14···C17^iii^	3.2418
C6···H18B	2.6342	H14···C20^iii^	2.9477
C7···H18B	2.9925	H14···H10^iv^	3.2613
C8···H5	2.6624	H14···H17^iii^	3.2271
C8···H10	2.6746	H14···H20^iii^	2.7166
C8···H14	3.2926	H15A···O3^vi^	3.5892
C8···H18A	3.3067	H15A···C10^iv^	3.3524
C8···H18B	2.8563	H15A···C17^iii^	2.8789
C9···H10	3.2716	H15A···C19^iii^	3.0330
C10···H9	3.2726	H15A···C20^iii^	3.3691
C12···H5	2.5989	H15A···H10^iv^	3.0153
C13···H15A	2.6653	H15A···H17^iii^	3.0194
C13···H15B	2.7295	H15A···H19^iii^	3.2603
C13···H19	3.2485	H15B···Cl2^vi^	3.3943
C13···H20	3.2564	H15B···O3^vi^	2.6846
C14···H15A	2.5490	H15B···O4^ii^	2.8852
C14···H15B	3.1763	H15B···C10^ii^	3.2487
C14···H18A	2.5431	H15B···C18^vi^	3.4691
C14···H18B	2.9566	H15B···H10^ii^	2.5476
C14···H20	3.0445	H15B···H17^iii^	3.5708
C15···H13	2.6087	H15B···H18A^vi^	3.1415
C15···H14	2.5581	H17···O3^v^	3.0772
C15···H20	2.7292	H17···N5^vii^	3.2276
C17···H22	3.2478	H17···C6^v^	3.5927
C18···H14	2.5802	H17···C8^v^	3.5062
C19···H13	3.2458	H17···C12^v^	3.1140
C19···H20	3.2642	H17···C14^v^	3.5246
C20···H5	3.1890	H17···C14^vii^	3.1469
C20···H13	3.2529	H17···C15^vii^	3.4792
C20···H14	3.5740	H17···C18^iv^	3.5334
C20···H15A	3.2015	H17···C18^v^	3.3747
C20···H15B	3.1408	H17···H14^vii^	3.2271
C20···H19	3.2661	H17···H15A^vii^	3.0194
C21···H5	2.9716	H17···H15B^vii^	3.5708
C21···H14	3.2721	H17···H18A^v^	3.3813
C21···H17	3.2625	H17···H18B^iv^	2.5793
C21···H22	3.2644	H17···H20^v^	3.4719
C22···H17	3.2473	H18A···O4^iv^	2.7604
H5···H14	2.7144	H18A···O4^iii^	2.8400
H5···H15A	2.7426	H18A···N5^iv^	2.9479
H5···H15B	2.2499	H18A···N5^iii^	3.5007
H5···H20	2.7282	H18A···C8^iii^	3.5501
H13···H15A	2.5495	H18A···C15^iv^	3.5736
H13···H15B	2.6719	H18A···H5^iv^	2.4038
H13···H22	2.3245	H18A···H5^iii^	2.8905
H14···H15A	2.3613	H18A···H15B^iv^	3.1415
H14···H15B	3.4285	H18A···H17^v^	3.3813
H14···H18A	2.2803	H18A···H20^iii^	2.8599
H14···H18B	3.0427	H18B···O4^iii^	3.4985
H14···H20	3.2864	H18B···C17^vi^	2.6983
H15A···H20	3.4440	H18B···C19^vi^	3.3900
H15B···H20	3.3569	H18B···C20^vi^	3.0242
H17···H19	2.3321	H18B···C20^iii^	3.5530
H17···H20	2.3436	H18B···H5^iii^	3.5998
H19···H22	2.3289	H18B···H17^vi^	2.5793
Cl1···H9^i^	3.1181	H18B···H20^vi^	3.1266
Cl1···H13^vi^	3.5668	H18B···H20^iii^	2.6531
Cl1···H13^ii^	3.2600	H19···Cl1^xii^	3.5454
Cl1···H19^ix^	3.5454	H19···Cl2^v^	3.5181
Cl1···H22^ii^	3.4083	H19···O3^v^	2.8986
Cl1···H22^ix^	2.8136	H19···C6^v^	2.7245
Cl2···H9^viii^	3.4352	H19···C7^v^	3.2656
Cl2···H15B^iv^	3.3943	H19···C16^v^	2.9361
Cl2···H19^v^	3.5181	H19···H15A^vii^	3.2603
Cl2···H22^x^	3.3865	H20···O4^ii^	3.5806
O3···H5^iv^	3.4434	H20···C12^vii^	3.3837
O3···H15A^iv^	3.5892	H20···C14^vii^	3.1337
O3···H15B^iv^	2.6846	H20···C18^vii^	3.0867
O3···H17^v^	3.0772	H20···H14^vii^	2.7166
O3···H19^v^	2.8986	H20···H17^v^	3.4719
O4···H5^ii^	2.4314	H20···H18A^vii^	2.8599
O4···H14^vi^	2.6862	H20···H18B^iv^	3.1266
O4···H15B^ii^	2.8852	H20···H18B^vii^	2.6531
O4···H18A^vi^	2.7604	H20···H20^v^	3.5598
O4···H18A^vii^	2.8400	H22···Cl1^ii^	3.4083
O4···H18B^vii^	3.4985	H22···Cl1^xii^	2.8136
O4···H20^ii^	3.5806	H22···Cl2^xi^	3.3865
N5···H17^iii^	3.2276	H22···C9^iv^	3.2255
N5···H18A^vi^	2.9479	H22···C16^iv^	3.3726
N5···H18A^vii^	3.5007	H22···H9^iv^	3.2522
C6···H17^v^	3.5927	H22···H9^xii^	3.4450
			
C6—O3—C18	112.97 (12)	C15—C21—C20	123.44 (16)
C14—N5—C15	122.12 (14)	C13—C22—C19	120.12 (19)
O3—C6—C7	122.55 (13)	C14—N5—H5	118.942
O3—C6—C16	118.21 (14)	C15—N5—H5	118.939
C7—C6—C16	119.16 (14)	C11—C9—H9	120.638
C6—C7—C8	119.51 (13)	C16—C9—H9	120.643
C6—C7—C10	119.71 (14)	C7—C10—H10	120.227
C8—C7—C10	120.61 (14)	C11—C10—H10	120.204
O4—C8—C7	120.75 (14)	C21—C13—H13	119.676
O4—C8—C12	124.53 (14)	C22—C13—H13	119.686
C7—C8—C12	114.69 (14)	N5—C14—H14	116.629
C11—C9—C16	118.72 (16)	C12—C14—H14	116.629
C7—C10—C11	119.57 (14)	N5—C15—H15A	108.564
Cl1—C11—C9	118.55 (12)	N5—C15—H15B	108.559
Cl1—C11—C10	119.79 (13)	C21—C15—H15A	108.561
C9—C11—C10	121.67 (15)	C21—C15—H15B	108.557
C8—C12—C14	122.53 (15)	H15A—C15—H15B	107.537
C8—C12—C18	117.64 (15)	C19—C17—H17	119.888
C14—C12—C18	119.47 (15)	C20—C17—H17	119.880
C21—C13—C22	120.64 (16)	O3—C18—H18A	109.152
N5—C14—C12	126.74 (16)	O3—C18—H18B	109.161
N5—C15—C21	114.83 (14)	C12—C18—H18A	109.151
Cl2—C16—C6	119.12 (13)	C12—C18—H18B	109.152
Cl2—C16—C9	119.72 (13)	H18A—C18—H18B	107.860
C6—C16—C9	121.15 (15)	C17—C19—H19	120.063
C19—C17—C20	120.23 (18)	C22—C19—H19	120.065
O3—C18—C12	112.27 (13)	C17—C20—H20	120.039
C17—C19—C22	119.87 (18)	C21—C20—H20	120.047
C17—C20—C21	119.91 (17)	C13—C22—H22	119.943
C13—C21—C15	117.33 (15)	C19—C22—H22	119.938
C13—C21—C20	119.22 (15)		
			
C6—O3—C18—C12	−51.66 (17)	C7—C10—C11—C9	−0.2 (3)
C6—O3—C18—H18A	−172.8	H10—C10—C11—Cl1	−0.5
C6—O3—C18—H18B	69.5	H10—C10—C11—C9	179.8
C18—O3—C6—C7	29.69 (19)	C8—C12—C14—N5	0.6 (3)
C18—O3—C6—C16	−153.42 (12)	C8—C12—C14—H14	−179.4
C14—N5—C15—C21	89.40 (18)	C8—C12—C18—O3	43.1 (2)
C14—N5—C15—H15A	−32.3	C8—C12—C18—H18A	164.3
C14—N5—C15—H15B	−148.9	C8—C12—C18—H18B	−78.0
C15—N5—C14—C12	175.36 (14)	C14—C12—C18—O3	−143.61 (15)
C15—N5—C14—H14	−4.6	C14—C12—C18—H18A	−22.5
H5—N5—C14—C12	−4.6	C14—C12—C18—H18B	95.2
H5—N5—C14—H14	175.4	C18—C12—C14—N5	−172.27 (14)
H5—N5—C15—C21	−90.6	C18—C12—C14—H14	7.7
H5—N5—C15—H15A	147.7	C21—C13—C22—C19	−0.0 (3)
H5—N5—C15—H15B	31.1	C21—C13—C22—H22	180.0
O3—C6—C7—C8	3.3 (3)	C22—C13—C21—C15	−178.49 (14)
O3—C6—C7—C10	178.56 (12)	C22—C13—C21—C20	0.6 (3)
O3—C6—C16—Cl2	2.8 (2)	H13—C13—C21—C15	1.5
O3—C6—C16—C9	−178.65 (12)	H13—C13—C21—C20	−179.4
C7—C6—C16—Cl2	179.76 (12)	H13—C13—C22—C19	180.0
C7—C6—C16—C9	−1.6 (3)	H13—C13—C22—H22	−0.0
C16—C6—C7—C8	−173.54 (13)	N5—C15—C21—C13	−174.97 (12)
C16—C6—C7—C10	1.7 (3)	N5—C15—C21—C20	6.0 (2)
C6—C7—C8—O4	165.29 (13)	H15A—C15—C21—C13	−53.3
C6—C7—C8—C12	−12.7 (2)	H15A—C15—C21—C20	127.7
C6—C7—C10—C11	−0.8 (3)	H15B—C15—C21—C13	63.3
C6—C7—C10—H10	179.2	H15B—C15—C21—C20	−115.7
C8—C7—C10—C11	174.38 (12)	C19—C17—C20—C21	0.2 (3)
C8—C7—C10—H10	−5.6	C19—C17—C20—H20	−179.8
C10—C7—C8—O4	−9.9 (3)	C20—C17—C19—C22	0.4 (3)
C10—C7—C8—C12	172.11 (13)	C20—C17—C19—H19	−179.6
O4—C8—C12—C14	−2.0 (3)	H17—C17—C19—C22	−179.6
O4—C8—C12—C18	171.05 (13)	H17—C17—C19—H19	0.3
C7—C8—C12—C14	175.92 (13)	H17—C17—C20—C21	−179.8
C7—C8—C12—C18	−11.05 (19)	H17—C17—C20—H20	0.2
C11—C9—C16—Cl2	179.25 (12)	C17—C19—C22—C13	−0.5 (3)
C11—C9—C16—C6	0.7 (3)	C17—C19—C22—H22	179.5
C16—C9—C11—Cl1	−179.46 (13)	H19—C19—C22—C13	179.5
C16—C9—C11—C10	0.3 (3)	H19—C19—C22—H22	−0.5
H9—C9—C11—Cl1	0.5	C17—C20—C21—C13	−0.7 (3)
H9—C9—C11—C10	−179.7	C17—C20—C21—C15	178.32 (14)
H9—C9—C16—Cl2	−0.8	H20—C20—C21—C13	179.3
H9—C9—C16—C6	−179.3	H20—C20—C21—C15	−1.7
C7—C10—C11—Cl1	179.54 (12)		

Symmetry codes: (i) *x*, −*y*+3/2, *z*+1/2; (ii) −*x*+1, −*y*+1, −*z*+1; (iii) *x*−1/2, *y*, −*z*+1/2; (iv) −*x*+1/2, −*y*+1, *z*−1/2; (v) −*x*+1, −*y*+1, −*z*; (vi) −*x*+1/2, −*y*+1, *z*+1/2; (vii) *x*+1/2, *y*, −*z*+1/2; (viii) *x*, −*y*+3/2, *z*−1/2; (ix) −*x*+1, *y*+1/2, −*z*+1/2; (x) −*x*+1/2, *y*+1/2, *z*; (xi) −*x*+1/2, *y*−1/2, *z*; (xii) −*x*+1, *y*−1/2, −*z*+1/2.

### Hydrogen-bond geometry (Å, º)

**Table:**

*D*—H···*A*	*D*—H	H···*A*	*D*···*A*	*D*—H···*A*
N5—H5···O4	0.88	2.15	2.7737 (19)	127

## References

[bb1] Benaouida, M. A., Chetioui, S. & Bouaoud, S. E. (2013). *Acta Cryst.* E**69**, o867–o868. 10.1107/S1600536813012245 PMC3685028 23795047

[bb2] Khan, K. M., Ambreen, N., Hussain, S., Perveen, S. & Choudhary, M. I. (2009). *Bioorg. Med. Chem.* **17**, 2983–2988. 10.1016/j.bmc.2009.03.020 19329330

[bb3] Małecka, M. & Budzisz, E. (2006). *Acta Cryst.* E**62**, o5058–o5060.

[bb4] North, A. C. T., Phillips, D. C. & Mathews, F. S. (1968). *Acta Cryst.* A**24**, 351–359.

[bb5] Rigaku (1999). *WinAFC Diffractometer Control Software* Rigaku Corporation, Tokyo, Japan.

[bb6] Rigaku (2010). *CrystalStructure* Rigaku Corporation, Tokyo, Japan.

[bb7] Sheldrick, G. M. (2008). *Acta Cryst.* A**64**, 112–122. 10.1107/S0108767307043930 18156677

[bb8] Tu, Q. D., Li, D., Sun, Y., Han, X. Y., Yi, F., Sha, Y., Ren, Y. L., Ding, M. W., Feng, L. L. & Wan, J. (2013). *Bioorg. Med. Chem.* **21**, 2826–2831. 10.1016/j.bmc.2013.04.003 23623712

